# Historical geospatial dataset of Cyprus from British administration maps of the 19th century

**DOI:** 10.1016/j.dib.2024.111084

**Published:** 2024-10-29

**Authors:** Evangelos Papadias, Kleomenis Kalogeropoulos, Christos Polykretis, Athanasios Psarogiannis, Georgios Chalkias, Christos Chalkias

**Affiliations:** a Geography Department, School of Environment, Geography and Applied Economics, Harokopio University of Athens, Kallithea, Athens, Attica, Greece; b Department of Surveying and Geoinformatics Engineering, University of West Attica, Aigaleo, Attica, Greece

**Keywords:** HGIS, Roads, Hydrography, Land cover, Population

## Abstract

The geospatial dataset presented in this manuscript, represents elements of the historical, natural, political and cultural environment of Cyprus, as it was recorded by British officials during the early years (1878–1883) of their administration on the island. The data were derived from Horatio Herbert Kitchener's map of Cyprus, published in 1885 in 15 sheets, at a scale of 1:63 360 (1 inch to 1 mile), which is considered a milestone in the cartography of the island. The geospatial layers were extracted my manual on-screen digitization of the geographic features depicted on the georeferenced seamless mosaic created by the individual sheets (Chalkias et al., 2023). The database consists of the following vector layers: (a) road network, (b) hydrographic network, (c) administration boundaries as well as land cover/use in raster format and covers the entire area of the island (9 247 km^2^). In addition, the locations and the population attributes recorded in the census of 1881, have been added to the database as well. Historical maps and censuses are a valuable and the most common source of geospatial data used in historical research, when space and the spatial distribution of phenomena, are considered a critical parameter. However, among other, the availability of historical geospatial data, is recognized as a limitation to the further development of Historical GIS. Therefore, this dataset aims to contribute to this need.

Specifications Table

**Table:** 

Subject	Earth and Planetary Sciences: Geographical Information System.
Specific subject area	Historical GIS applications, Historical Geography.
Type of data	.shp (ESRI shapefile with attributes) .tif (single band raster layer)
Data collection	The dataset layers were extracted by manual vectorization using GIS-based digitization procedures, from the georeferenced sheets of Kitchener's map [1]. All spatial processes were carried out using ArcGIS 10.6.1 and the results were stored originally inside a file geodatabase. Population data were manually extracted from the published printed tables [2] and organized in tabular form using .csv file format. Record matching with their corresponding locations digitized on the map, was performed manually. ArcGIS Pro 3.2.2 was used for the curation of the final dataset.
Data source location	The original data were collected in Cyprus during the period 1878-1883. Digital data derived from the original data, were created at the Department of Geography, Harokopio University of Athens, Greece.
Data accessibility	Repository name: Mendeley Data Data identification number: 10.17632/dvvm9cns2x.2 Direct URL to data: https://data.mendeley.com/datasets/dvvm9cns2x/2
Related research article	C. Chalkias, E. Papadias, C. Vradis, C. Polykretis, K. Kalogeropoulos, A. Psarogiannis, G. Chalkias, Developing and Disseminating a New Historical Geospatial Database from Kitchener's 19th Century Map of Cyprus, ISPRS International Journal of Geo-Information 12 (2023) 74. https://doi.org/10.3390/ijgi12020074.

## 1 Value of the Data

• The dataset includes various elements of the historical, natural, political and cultural environment of Cyprus as recorded by British officials during the early years of its administration. Kitchener's trigonometrical map, is the largest scale survey and the only source that provides professionally surveyed historical geographic data for Cyprus in the 19th century, at this level of historical accuracy and detail.
• Fills a critical gap in the availability of historical geospatial data that is often a limitation in historical GIS research. Provides valuable data to advance the field of historical GIS and support further research and development.
• The dataset is useful for historical research, archeology, environmental studies, urban planning and heritage conservation. Provides detailed historical cartographic information and integrates places and population attributes, adding a demographic dimension to the geospatial data. Enables a detailed analysis of the spatial distribution and changes of historical phenomena and facilitates studies of population distribution and demographic changes over time.
• By compiling and digitizing these historical elements, the dataset not only preserves valuable historical information, but also makes it accessible to modern analysis techniques, bridging the gap between historical cartography and modern GIS applications.
• The dataset covers the entire area of Cyprus (9 247 km²) and enables comprehensive spatial analysis of the entire island. It can be coupled with other more modern historical datasets of similar scale [3].

## 2 Background

The arrival of the British in Cyprus in 1878 marked a turning point in the development of the island's natural, political and cultural landscape. Ottomans ruled Cyprus since 1571 after military conquest. However, the administration of the island of Cyprus, was handed over peacefully by a treaty signed between the British and the Ottoman Empire, in the context of the congress of Berlin (July 1878). Surveying the new region for administration purposes was a high priority for the British, therefore a population census and a large-scale mapping of the island were finished by the end of 1883. The population census of 1881 and Kitchener's map published in 1885, encapsulate the physical and cultural landscape of the late Ottoman era. Additionally, the map contains the early intentions of the British to establish administration over the island. The motivation behind compiling the dataset presented here, was to provide geospatial data to historians, environmental scientists and relative academics, that allows a more comprehensive study of Cyprus's landscape and geography in the 19th century. The limited availability of historical geospatial data is usually a significant obstacle to such research [4]. Additionally, the dataset adds value to previous work [1], by providing essential geospatial layers to the academic community for further analysis.

## 3 Data Description

**Fig. 1:**
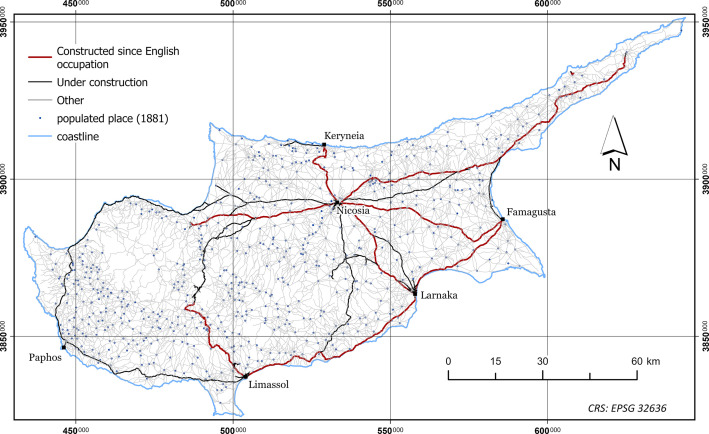
The road network according to Kitchener's map legend.

**Fig. 2:**
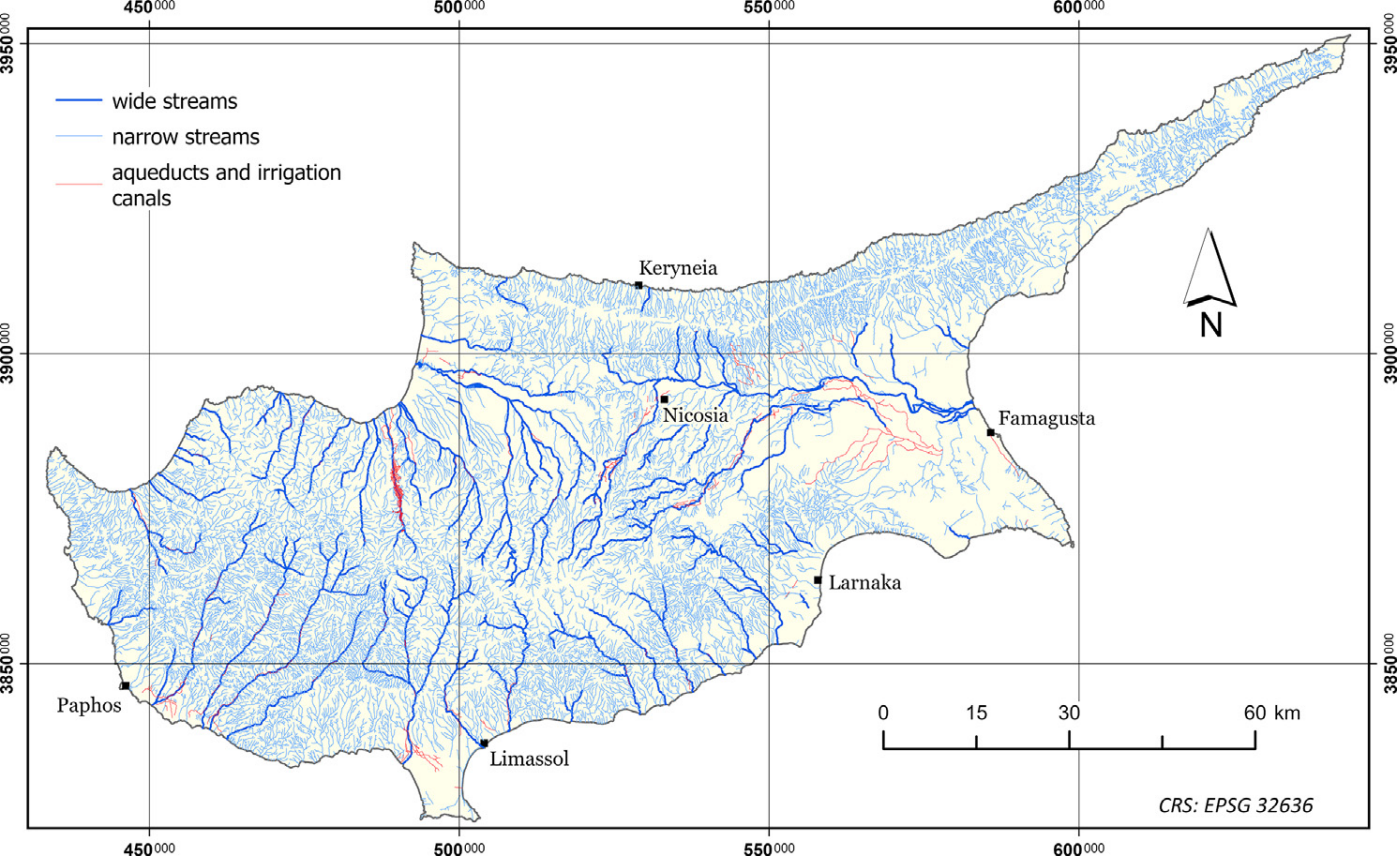
The stream network as symbolized on the map.

**Fig. 3:**
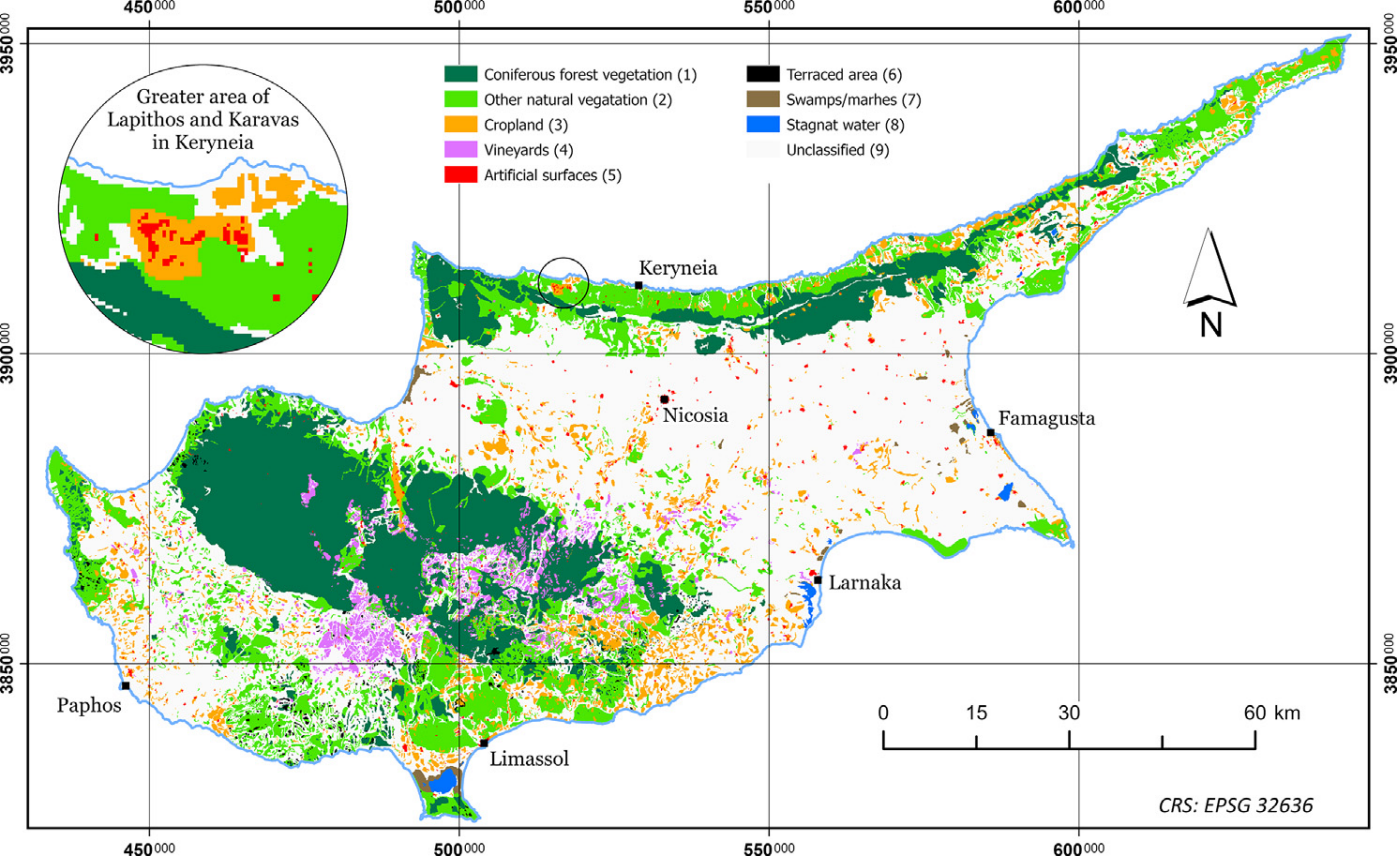
The land cover/use according to Kitchener's map.

**Fig. 4:**
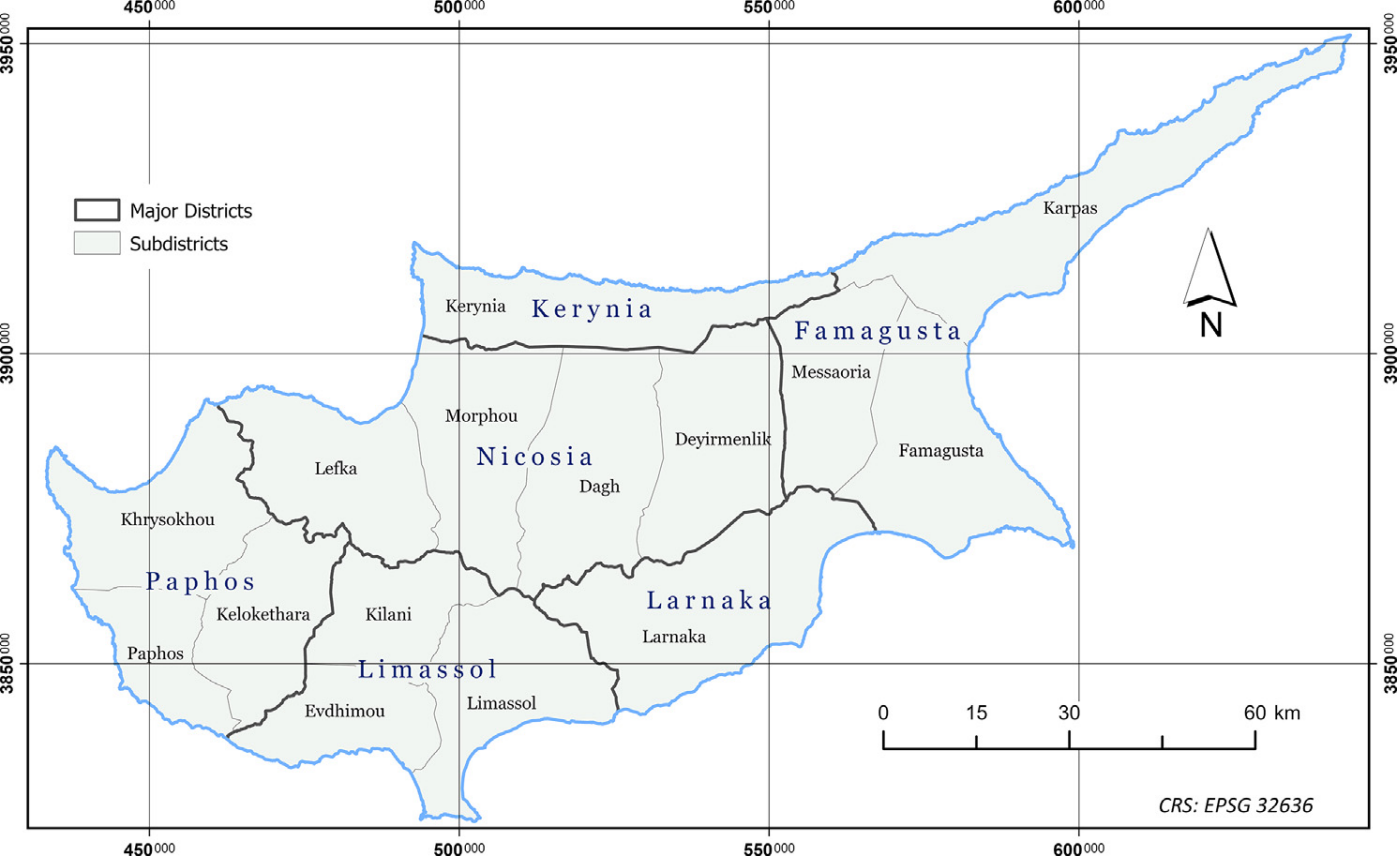
The two levels of administration and the coastline. Names have been recorded as found written on the map.

The dataset presented in this paper consists of six geospatial layers; the coastline (Fig. 4), the administration boundaries (Fig. 4), the road network (Fig. 1), the hydrographic network (Fig. 2) and the land cover/use (Fig. 3), all derived from Kitchener's map of Cyprus [5] and additionally, the population attributes from the census of 1881 [2]. The features of the geospatial layers have been organized in polyline, point and polygon ESRI shapefile format and a single band raster layer. The geometry of the layers corresponds to the Coordinate Reference System EPSG: 32636 (WGS 84 / UTM zone 36N). Necessary metadata has been embedded in the published layers.

### 3.1 Road Network

The road_network_1885 layer includes 19 695 polyline features and two numeric fields in its attribute table; class and Length in meters. The typology of the class field is explained in Table 1. All features have been classified into three distinct categories according to the map's legend.

**Table 1:** Summary of the road classes included in the road network layer, according to the map's legend.

Class	Description	features	Length [km]	% of total
1	Other	18 267	14 288.22	93.66
2	Under construction	813	525.42	3.44
3	Constructed since English occupation	615	442.61	2.90
	Total	19 695	15 256.25	100

### 3.2 Hydrographic Network

The stream_network_1885 layer includes 16 971 polyline features and two numeric fields in its attribute table; type and Length in meters. All features have been classified making the least possible interpretation assumptions, into three categories using the explanations given for each feature on the map and the typology of the line-type symbolization scheme. Further explanations are given in the Experimental Design, Materials and Methods section. The typology of the type field is explained in Table 2.

**Table 2:** Summary of the categories of the hydrographic network, according to the symbolization scheme used for the map.

Type	Description	Features	Length [km]	% of Total
1	Wide streams	445	1 592.16	9.58
2	narrow streams	15 769	14 448.77	86.94
3	Aqueducts and irrigation canals	757	577.73	3.48
	Total	16 971	16 618.66	100

### 3.3 Land cover

Land cover layer (land_cover_1885.tif) is a single band raster layer (2 105 columns and 1 269 rows) with 9 values. The shape of the cell is square and its size is 100 meters. Typology is explained in Table 3.

**Table 3:** Summary of the typology of the values in land cover/use raster layer.

Value	Description	Cells	% of Total
1	Coniferous forest vegetation	180 124	19.48
2	Other natural vegetation	152 032	16.44
3	Cropland	51 644	5.58
4	Vineyards	23 826	2.58
5	Artificial surfaces	6 614	0.72
6	Terraced area	3 533	0.38
7	Swamps/marshes	2 849	0.31
8	Stagnat water	2 648	0.29
9	Unclassified	501 388	54.22
	Total	924 658	100

### 3.4 Administrative Boundaries and Coastline

The administrative division layer (administration_1885.shp), includes 15 polygon features (subdistricts), a numeric field with an object ID and two textual fields in its attribute table with the relevant information about the name of each subdistrict and the name of the greater district each one belongs to. Names are set as they appear on the map. The coastline layer (coastline_1885.shp) contains a single polyline feature representing the coast of the island as it appears on the map (Fig. 4).

### 3.5 Populated Places

The populated places layer (census_1881.shp) includes 692 point features (Fig. 1) and six fields in its attribute table (Table 4)

**Table 4:** The description of the fields in the census_1881 layer's attribute table.

Field	Description	Type
District	First level of administration	String
Subdistrict	Second level of administration	String
name_cens	The placename as found written	String
Males	Total male population	Numeric
Females	Total female population	Numeric
Total	Total population	Numeric

## 4 Experimental Design, Materials and Methods

**Fig. 5:**
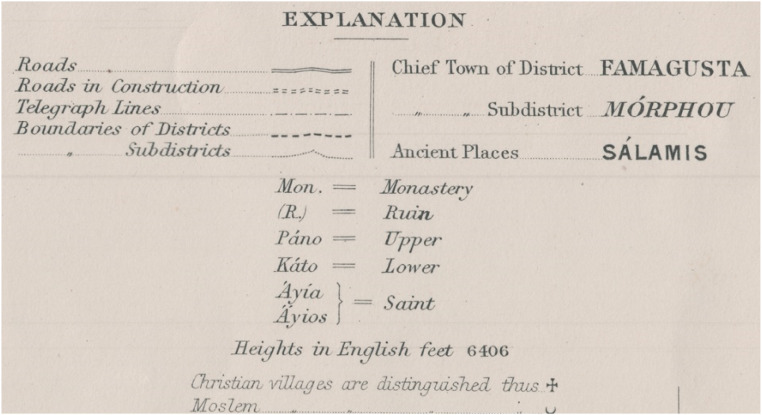
The legend included on the index sheet of Kitchener's map. An extended legend can be found in [7].

Kitchener's map was published in 1885 in 15 sheets, at a scale of 1:63 360 (one inch to one mile) and covers the entire island of Cyprus. It is the outcome of a trigonometrical survey initiated in September 1878 for which all field work and most of the office work had been completed by the end of 1882 [6]. Although the map contains a multitude of geographic features, the lack in detail on the legend of the map is noteworthy (Fig. 5). Therefore, we have tried to minimize the interpretation assumptions of the features depicted on the map in order to avoid misleading information. The map can be accessed dynamically through a geoportal [7].

The geospatial layers included in this dataset were produced by manually digitizing features on the georeferenced seamless mosaic of the 15 sheets created previously [1]. The original digitization was completed on screen with a fixed scale (1:5 000) using ArcGIS Desktop 10.6.1 for the process. For the final curation of the data, we used ArcGIS Pro 3.2.2. All layers have been checked for common topological errors such as gaps and overlapping features and additionally the streams and roads network have been checked for connectivity issues.

**Fig. 6:**
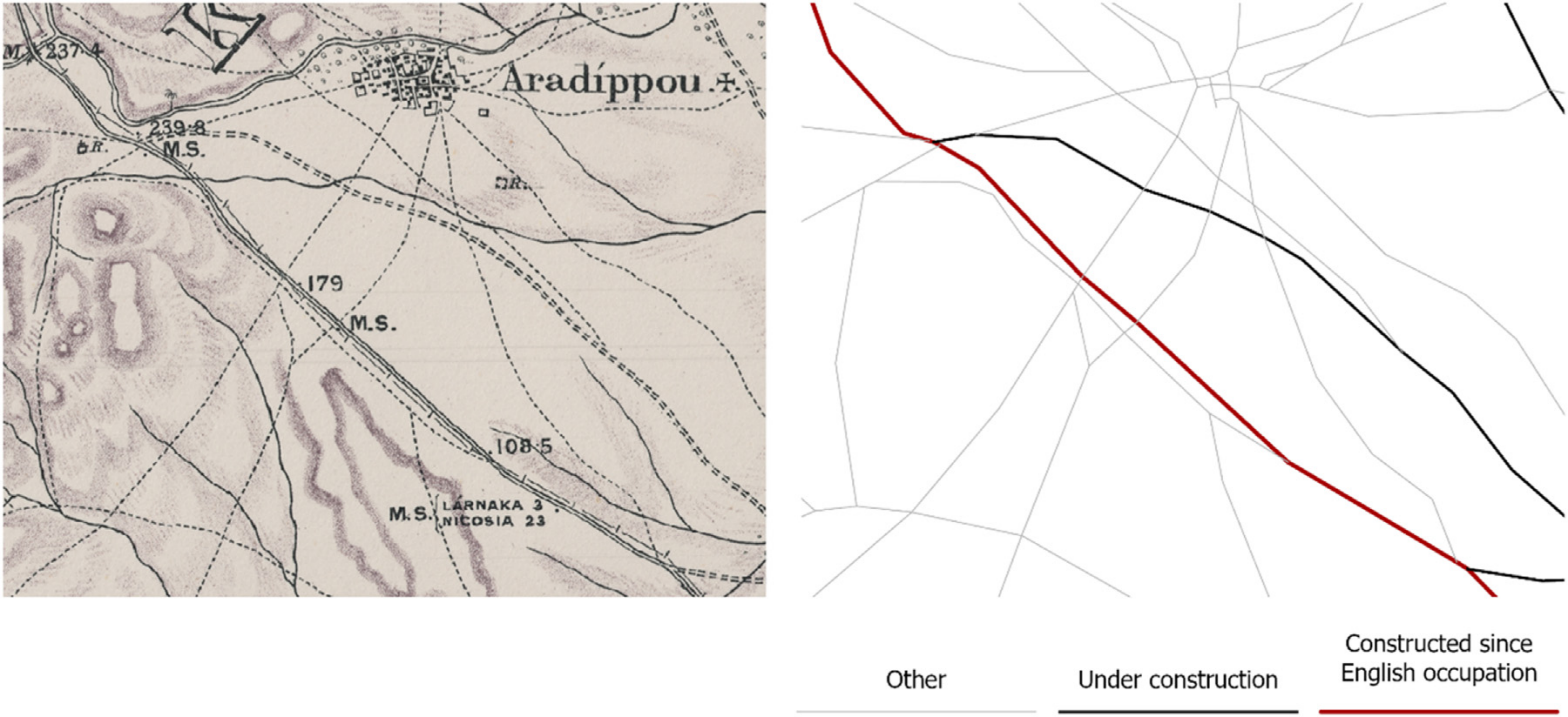
Detail from Kitchener's map sheet No. 10 (scale 1:50 000).

The road network has been classified into three categories following the explanations given in the map's legend (Fig. 5). Only two symbols are included in the road typology: Constructed since English occupation and Under construction, therefore the rest have been classified as Other. The symbol used by the compiler of the map for other roads is a single dashed line (Fig. 6) which according to the typology explained in other map legends of the same period in other areas surveyed by the same mapping services, corresponds to rural roads, cart-tracks and paths [8].

**Fig. 7:**
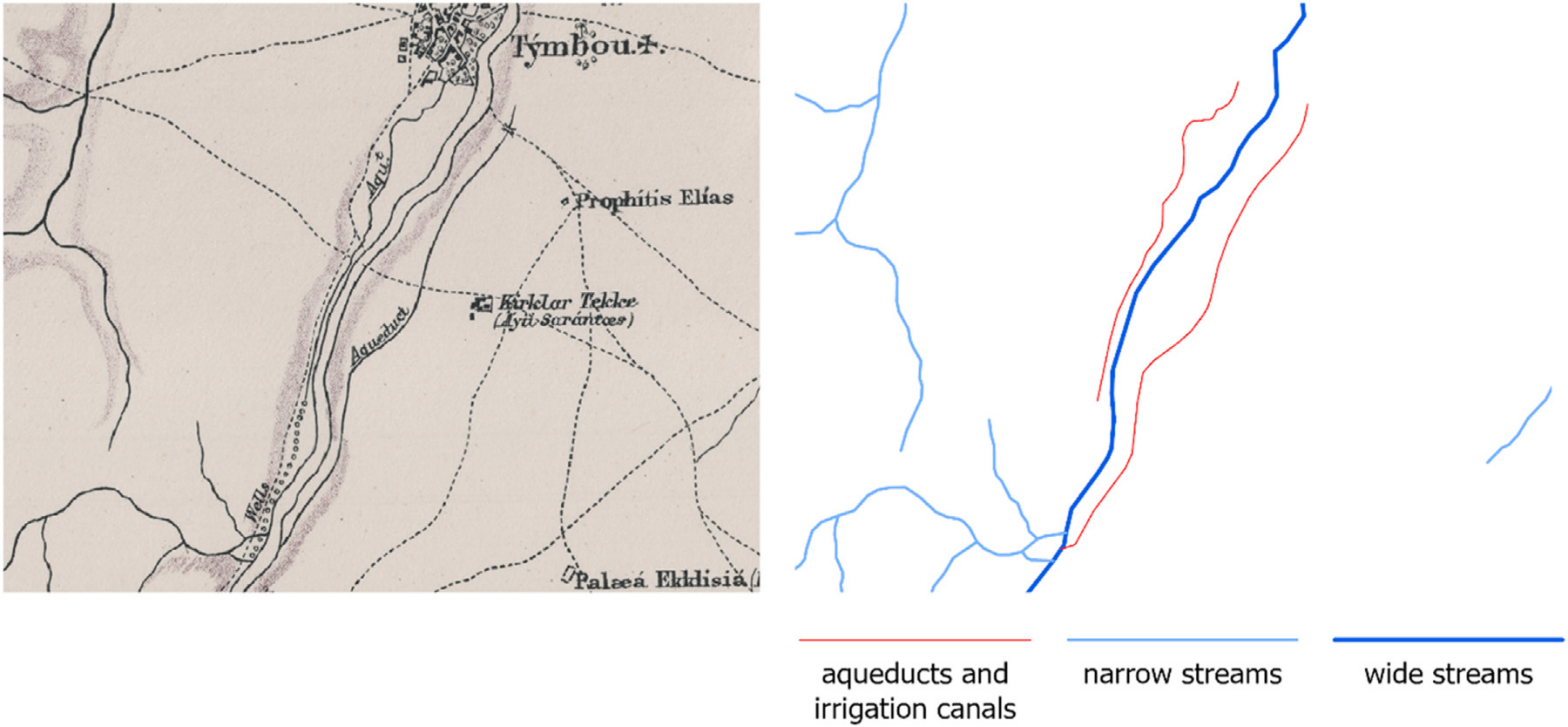
Detail from Kitchener's map sheet No. 10 (scale 1:50 000).

Explanations about the hydrographic features symbols used on the map are not included in the legend. However, three primary symbols are clearly distinguished: a double line of irregular thickness for the wider streams, a single solid line for narrower water courses and a solid line marked with the inscription “Aqueduct” or “Aqu.^t^” for aqueducts and irrigation canals (Fig. 7). All hydrographic features have been digitized downstream.

**Fig. 8:**
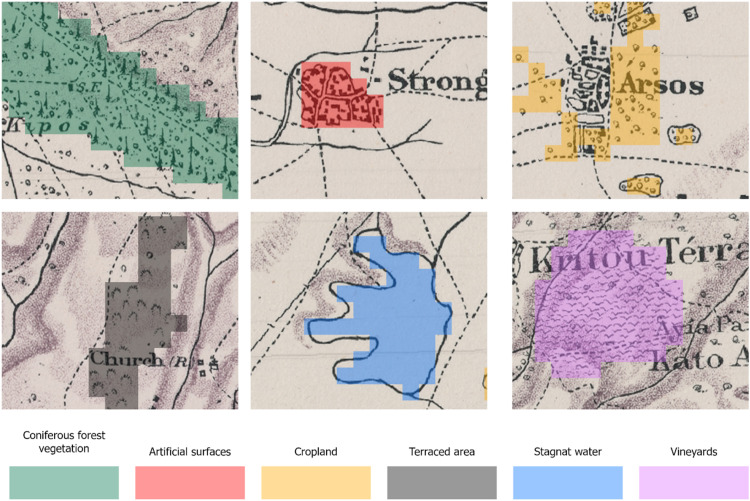
Detailed examples of the land cover/use raster layer (scale 1:25 000).

About the land cover/use features, the map's poor legend provides explicit information only for the vineyards. However, the sharp pictorial iconographic symbol used, leaves no doubt that describes pine trees (Fig. 8). The same applies for swamps/marshes and terraced areas as well as water bodies which are clearly and easily recognizable on the map. Artificial surfaces were determined by the polygon containing dense human constructions around settlements. Other cropland is distinguishable by the ordered arrangement of the used symbols. Kitchener's map for 54 % of the island's surface provides no information at all. However, examining older as well as newer maps and surveys, we have found that annual crops, such as cereals and cotton, bare soil and grassland were included in the Unclassified category of the land cover/use layer provided in this dataset. The original vector layer was converted to a grid of square cells (100 m cell-size).

The map includes two levels of administrative division. The first level concerns the six large provinces and the second their subdivisions into smaller regions following the administration model of the late Ottoman era (*nahiye*). An examination of the content of the first two British population censuses, carried out in 1881 and 1891, revealed that the administrative boundaries shown on Kitchener's map published in 1885, correspond to the census of 1891 and not to 1881. Τhe administrative division resulting from the 1881 census seems to agree with the administrative boundaries of the late Ottoman era found on maps of that period [9].

The population data recorded in the census of Cyprus, 1881, have been extracted manually from the final report published in 1884 [2] and were organized in tabular format. The population census carried out in 1881 was the first of a series of modern statistical measuring of the population, entailed by the British and the first official enumeration of the total population of the island of Cyprus. The record enlists the inhabitants in 707 locations out of which 692 were matched with locations digitized from Kitchener's map using their names. 359 individuals (0.19 % of the total population) in 15 records were not matched with any location on the map and have been omitted from the dataset published in this article.

## Limitations

Precision and accuracy of the content in historical documents should always be under investigation. Spatial data created by historical maps and other sources, inevitably carry all the limitations of the original source. Furthermore, errors and limitations are introduced during the digitization process [10]. The precision of the data is discussed in previous work [1] and for the accuracy of the content it should be considered that the map represents the interpretation of the landscape by the map creator/creators and that contains only the elements of their interest.

## Ethics Statement

The authors declare that the hereby presented data and data article fully comply with the Journal's policy regarding authors’ duties, data integrity, and experimental requirements. The study does not involve experiments on humans or animals and no data was collected from social media platforms.

## CRediT authorship contribution statement

**Evangelos Papadias:** Conceptualization, Methodology, Software, Data curation, Writing – original draft, Visualization, Investigation, Validation, Writing – review & editing. **Kleomenis Kalogeropoulos:** Data curation, Investigation, Validation. **Christos Polykretis:** Data curation, Investigation, Validation. **Athanasios Psarogiannis:** Data curation, Investigation, Validation. **Georgios Chalkias:** Data curation, Investigation, Validation. **Christos Chalkias:** Conceptualization, Methodology, Writing – original draft, Supervision, Funding acquisition, Project administration, Writing – review & editing.

## Data Availability

Mendeley DataGeospatial dataset from Kitchener's Map of Cyprus (Original data).

## References

[1] Chalkias C., Papadias E., Vradis C., Polykretis C., Kalogeropoulos K., Psarogiannis A., Chalkias G. (2023). Developing and disseminating a new historical geospatial database from Kitchener's 19th century map of Cyprus. ISPRS Int. J. Geoinf..

[2] F. Barry, Report on the census of Cyprus 1881: with appendix, eyre and spottiswoode, London, 1884.

[3] Papadias E., Chalkias C. (2023). A historical geospatial database of the island of Cyprus in the 1960s. Data Br..

[4] Gregory I.N., Healey R.G. (2007). Historical GIS: structuring, mapping and analysing geographies of the past. Prog. Hum. Geogr..

[5] H.H. Kitchener, A trigonometrical survey of the Island of Cyprus, (1885).

[6] R. Shirley, Kitchener's survey of Cyprus 1878-1883: the full triangulated survey and mapping of the Island, The Bank of Cyprus Cultural Foundation, Nicosia, 2001.

[7] Anon, https://kitchener.hua.gr/en (accessed 13 April 2024).

[8] Levin N. (2006). The Palestine exploration fund map (1871–1877) of the holy land as a tool for analysing landscape changes: the coastal dunes of Israel as a case study. Cartogr. J..

[9] Anon. "The geographical magazine", Map of Cyprus 1878, (1878). https://sylviaioannoufoundation.org/el/collection/digital-library/m0751/.

[10] Gregory I. (2005).

